# MB-OFDM-UWB Based Wireless Multimedia Sensor Networks for Underground Coalmine: A Survey

**DOI:** 10.3390/s16122158

**Published:** 2016-12-16

**Authors:** Ruisong Han, Wei Yang, Kaiming You

**Affiliations:** School of Electronic and Information Engineering, Beijing Jiaotong University, Beijing 100044, China; hanruisong@bjtu.edu.cn (R.H.); youkaiming@bjtu.edu.cn (K.Y.)

**Keywords:** wireless multimedia sensor networks (WMSNs), underground coalmine, multi-band orthogonal frequency-division multiplexing (MB-OFDM), ultra-wide band (UWB), network management

## Abstract

Safety production of coalmines is a task of top priority which plays an important role in guaranteeing, supporting and promoting the continuous development of the coal industry. Since traditional wireless sensor networks (WSNs) cannot fully meet the requirements of comprehensive environment monitoring of underground coalmines, wireless multimedia sensor networks (WMSNs), enabling the retrieval of multimedia information, are introduced to realize fine-grained and precise environment surveillance. In this paper, a framework for designing underground coalmine WMSNs based on Multi-Band Orthogonal Frequency-Division Multiplexing Ultra-wide Band (MB-OFDM-UWB) is presented. The selection of MB-OFDM-UWB wireless transmission solution is based on the characteristics of underground coalmines. Network structure and design challenges are analyzed first, which is the foundation for further discussion. Then, key supporting technologies and open research areas in different layers are surveyed, and we provide a detailed literature review of the state of the art strategies, algorithms and general solutions in these issues. Finally, other research issues like localization, information processing, and network management are discussed.

## 1. Introduction

Safety production of coalmines is a task of top priority which plays an important role in guaranteeing, supporting and promoting the continuous development of coal industry. It is also a key part of the state work safety. Implementing the strategy of “Prospering the nation, the coal industry and the safety production by science” and establishing the long-term mechanism of coalmine safety production are essential for China’s coalmine industry [1].

In recent years, China has made considerable progress in coalmine safety production, but the situation is still severe which draws higher demands for the coalmine industry. To ensure the safe operation of coalmines and reduce the number of accidents and casualties to the maximum, the systems for coalmine monitoring, early warning and disaster relief have urgent demands for wireless video surveillance, wireless voice communication, underground environment monitoring and personnel positioning [2]. Video surveillance for belt conveyers and other removable heavy-duty manufacturing facilities, along with different kinds of temporary video monitoring tasks such as regional mine drainage surveillance, is an effective way to avoid accidents. Unfortunately, the existing Cable Monitoring System (CMS) cannot fully meet the requirements of these applications, due to the complex working conditions in underground mines and the limitations of the CMS. Thus, by using wireless method, the areas that are difficult to monitor for the CMS can be effectively, instantly and flexibly monitored.

Wireless sensor networks (WSNs), which have outstanding advantages of easy configuration, flexibility to shrink or expand the monitoring range, strong fault-tolerance and mobility, are going to play an important role in monitoring and analyzing the dynamic, hostile, unfamiliar and unexplored environment [2]. However, the monitoring environments in coalmine are very complicated and ever-changing, and simple scalar data collected by traditional WSNs cannot meet the requirements of comprehensive environment monitoring of underground coalmine, leading to a urgent need for the introduction of information-intensive multimedia content such as video and audio streams, and still images into the sensor network based monitoring tasks in coalmines.

On the basis of WSNs, wireless multimedia sensor networks (WMSNs) are new types of sensor networks which enable perception of multimedia content such as video and audio streams, and images [3,4,5]. The sensor nodes of WMSNs are usually equipped with inexpensive hardware such as complementary metal–oxide semiconductor (CMOS) cameras, microphones and other sensors with the ability to retrieve simple environment data. Compared with WSNs, WMSNs enable the retrieval of multimedia streams that are typically informative and can be used to realize fine-grained and precise environment surveillance. Therefore, it is an ideal and effective way to achieve multimedia surveillance on environments like underground coalmines [6].

Using WMSNs to monitor the environment of coalmines attracts interests of many researchers in recent years. For instance, strategies of deploying WMSNs in underground coalmine have been put forward by Han and Yang [7,8]. Sun et al. [9] proposed a wireless multimedia sensor network self-organization protocol for mine rescue that showed the schedules for establishing a self-organization network quickly and prolonging the lifetime of network. Zhang et al. [10] showed an ultra-wide band (UWB) transmitting and receiving strategy with complicated and low bit rate performances in different mine roadway environment. However, most of these works are theoretical research focusing on certain technology, from which we cannot get a comprehensive understanding of the overall structure and developments of WMSNs for underground coalmine.

In this article, a detailed survey about WMSNs for underground coalmine is conduct to discuss the main research challenges and the state of the art supporting technologies for the development of WMSNs for coalmine. The main purposes of our paper are twofold: to make a summary of the application status of WSNs and WMSNs in the underground coalmine, and to present a framework and related open issues for designing underground coalmine WMSNs based on MB-OFDM-UWB scheme. Since the MB-OFDM-UWB scheme has great potentials to promote the performance of the existing monitoring and communication system, we believe that our work can serve as a point of inspiration for readers wishing to start researching into this area or researchers who try to design innovative emergency communication and monitoring system.

The rest of this article is organized as follows. Section 2 introduces the overall design of WMSNs for underground coalmine. Section 3 discusses the key supporting technologies for WMSNs. After that, other research issues of WMSNs in coalmine scene are presented in Section 4. At last, Section 5 concludes this work.

## 2. Overall Design of the WMSNs for Underground Coalmine and Related Works 

### 2.1. Network Structure

According to demands for comprehensive monitoring, the wireless multimedia monitoring system for underground coalmine based on Multi-Band Orthogonal Frequency-Division Multiplexing UWB (MB-OFDM-UWB) WMSNs mainly provides the following functions: (1) video surveillance for underground environments; (2) wireless voice communication; (3) multi-parameter monitoring for underground coalmine; and (4) localization of sensor nodes and targets.

**Figure 1:**
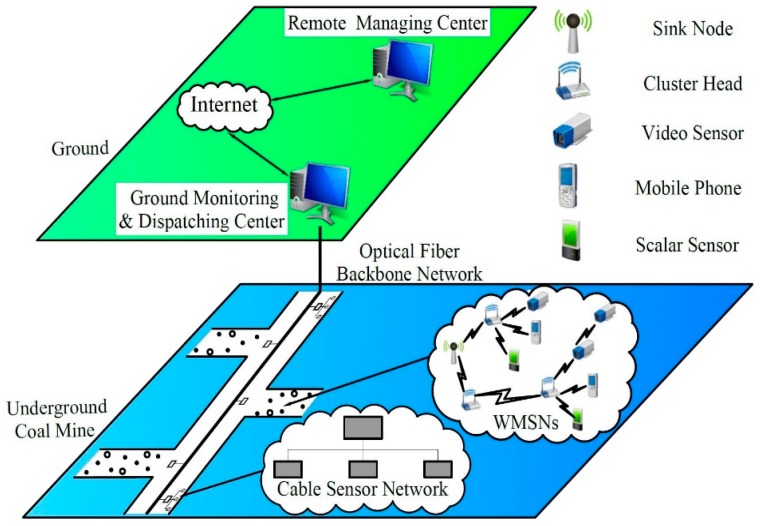
Network structure of the monitoring system for underground coalmines.

On the basis of the previous research and experiment works of Zhang et al. [11] and Han and Yang [7], the structure of the wireless multimedia monitoring system for underground coalmine based on MB-OFDM-UWB WMSNs is shown in Figure 1. Details about the network structure will also be discussed in this section.

As shown in Figure 1, WMSNs, consisting of wireless and interconnected smart devices such as cameras, phones and sensors, are mainly deployed in branch tunnels, and communicate with Ground Monitoring and Dispatching Center (GMDC) via optical fiber backbone network. Different from main tunnels’ landforms, landforms in some of the branch tunnels, mining areas and mined-out areas, are narrow and irregular. In this condition, WMSNs, as a great supplement to CMS and WSNs, are mainly deployed where the CMS is difficult to deploy, bringing in more flexibility and abilities of comprehensive surveillance. 

Specifically, for branch working zone, there are two basic methods of mining coalmines: longwall mining and room-and-pillar mining [12]. In longwall mining, coal is excavated by slicing along a long wall, and the mined-out areas are allowed to collapse. Basically, the longwall mining is a continuous operation involving using self-advancing hydraulic roof supports, coal-shearers, and armored conveyors. In contrast, the working space for room-and-pillar mining is laid out in a checkerboard of mining rooms and supporting pillars. For both mining methods, the working zones are dynamically changing. For instance, the roof of mined-out areas will collapse when hydraulic roof supports are removed, thus it is uneconomic and inconvenient to use a cable monitoring system that is stationary. Therefore, WMSNs are good alternative for achieving deploying flexibility and monitoring quality. Additionally, WMSN has obvious advantages in emergency applications such as disaster relief in virtue of simple networking, easy expansibility, and stable communication.

WMSNs are deployed in different local regions, and can be easily added in or removed off at any time according to varieties of monitoring requirements. Local regions have the characteristics of regional decentralization and small relevance to each other, and thus need to be monitored separately. Besides, monitoring contents and nodes configurations may vary dramatically. For instance, from the perspective of monitoring scale, the number of sensor nodes deployed in some local regions is small while that of other regions may be very large. Furthermore, the deployment of WMSNs for underground coalmine is first planned and fulfilled by workers, and that means it is a deterministic deployment instead of a random one which are usually used by WSNs. To ensure the safe operation of the WMSNs and the whole monitoring system, the system needs to be maintained and updated periodically.

Sensor nodes are the foundation of constructing WMSNs and all the protocols and algorithms concerning about sensor networks make sense when applied on sensor nodes. At present, the wireless sensor nodes designed or produced are generally classified into two categories: the first are nodes which take the general-purpose microprocessors as core components and use the design methods of embedded systems; the second are nodes designed using the platforms for designing specified device like FPGA and ASIC, such as PicoRadio [13], Multi-Radio WSN platform [14], and so on. Most sensor nodes are designed using the first method.

**Figure 2:**
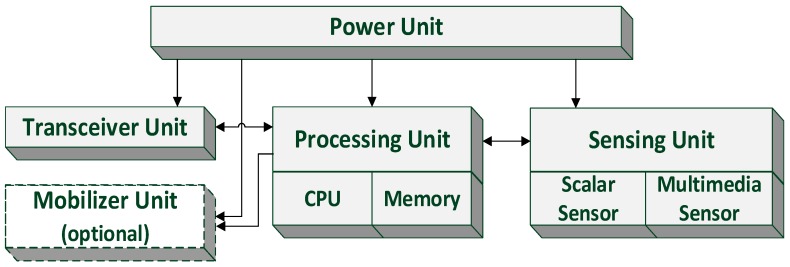
The components of a sensor node.

The difference between the hardware of sensor nodes in WMSNs and that in traditional WSNs is that the sensor nodes of WMSNs are equipped with CMOS sensors that can ubiquitously capture images and videos, and processors that have stronger processing capacities. However, the basic components of sensor nodes in WMSNs and those in WSNs are basically the same, which can be divided into four parts as shown in Figure 2: a sensing unit, a processing unit, a transceiver unit and a power unit [4,15]. The mobilizer unit in Figure 2, which allows movements or manipulation of objects or a sensor node itself, is optional according to the requirements for monitoring. Ignored by other surveys for WSNs and WMSNs, the electrical explosion proof issue for sensor networks is emphasized here, since the environment condition of a coalmine is usually harsh and dangerous and deserves more attention and design efforts. In addition, the issues of safety, immunity, protection, size, design, and reliability need to be considered for communication devices and equipment.

In the design of hardware, the prevalent technology of applying extended interfaces to connect sensors is used, which enhances the universality and flexibility of the hardware platform. From the perspective of processors, the sensor nodes can be divided into two categories. The first category uses the high-end processors represented by ARM, mostly supports dynamic voltage scaling (DVS) or dynamic frequency scaling (DFS), and has strong video processing capabilities. The Stargate [16] developed by Inter and manufactured by Crossbow is one of the typical examples of this category. In contrast, the other category, represented by Cyclops [17] based on Micaz platform, uses low-end processors that have low processing capabilities and could only finish the tasks of capturing simple images. 

The wireless transceiver unit uses 802.15.4 compliant Chipcon CC2420 radio, whose maximum transmission rate is 250 kbps, seeming powerless for transmitting video streams. Then, in some application scenarios that have high real-time requirements, 802.11b wireless cards are used. However, this increases the energy consumption and seems impractical. Further research work and techniques need to be introduced to solve the problem of high bandwidth demand and resource constraints.

With different sensing modules embedded into sensor nodes, variable parameters including temperature [18,19], humidity, gas and smoke concentration [11,18] and even seismic wave [20] can be collected. In addition, through UWB localization, which will be introduced later, we can also get the locations and movement trajectories of mines. Furthermore, with multimedia sensing modules, multimedia information like real-time videos and pictures of coalmines can also be acquired. The parameters that WMSNs can collect are not limited to these mentioned above. The schedule for choosing proper sensing units is based on the requirements of monitoring systems, and those sensing units should be compatible with other units.

### 2.2. Main Challenges for Designing

In recent years, the techniques of applying WSN into underground coalmines have been investigated. In [21,22], multi-parameters monitoring and early warning systems for coalmine environment based on mesh structured WSN and hierarchy WSN were proposed by Yang, making the integrated monitoring of gas, coal dust, temperature and so on come true. In these works, the mode of multi-parameters collection, i.e., periodic monitoring routine combined with interrupt service, was proposed. The supporting techniques such as routing mechanism, principles of slot allocation for wireless end device and data aggregation were also designed. A routing method, combing least-hop routing protocol, which is remaining energy aware, and tree protocol, was proposed. Besides, node address recycling strategy was also proposed. The experiment results carried out both in the laboratory and underground coalmine show that the designed system can monitor coalmine environment parameters effectively. Parts of the system functions have been applied in the actual projects [11]. However, the data transfer rate of the system is very low and only some simple scalar parameter can be collected. Thus, our work helps consider to use UWB to enhance the performance of existing monitoring systems. Other researchers have also contributed a lot in different directions of this area. Their work can be referred in [18,23,24]. 

At present, as mentioned in the previous section, the research work and application of WSN for underground coalmine are mainly based on ZigBee protocol in IEEE 802.15.4 standard. The basic requirement of the data rate for transferring CIF video streams is still higher than the date rate of ZigBee. Obviously, the maximum transmission rate of ZigBee could not satisfy the higher requirements of conducting multimedia surveillance in coalmine. On the other side, introducing Wi-Fi into coalmine seems a good choice for performing video surveillance, taking the advantages that Wi-Fi has a long transmission distance and high data rates [25]. Nevertheless, the high power consumption and poor multipath performance cannot guarantee the lifetime and transmission performance of sensor nodes. Therefore, UWB is a good solution which achieves good tradeoffs between diverse network performances. 

**Figure 3:**
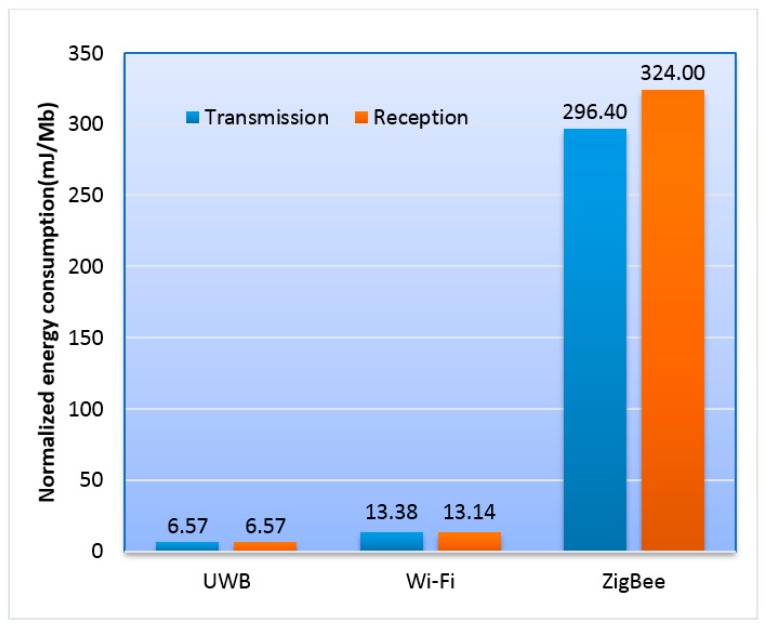
Comparison of UWB, Wi-Fi and ZigBee in normalized energy consumption by choosing four typical wireless products from [27].

A comparison among UWB, Wi-Fi, and ZigBee from the aspects of typical transmission distance, frequency, data rate, power consumption and complexity is presented in Table 1 [26,27,28]. As shown in the table, UWB outperforms Wi-Fi and ZigBee in data rate and normalized energy, which means UWB suffices for providing capabilities to transfer video stream with the resolution of even 1080p (1920 × 1080 pixel) while guaranteeing great energy consumption efficiency. Although the maximum transmission distance of UWB is not as long as the other two, we can actually deploy more sensor nodes in the network to make up the deficiency and to provide more flexibility for the environment like the room-and-pillar working zone. In Figure 3, the normalized energy consumption [27] of the three candidates in shown. As evident from this figure, UWB has great advantages in the normalized energy consumption which indicates that UWB is more efficient when transferring a large amount of multimedia information. In addition, except for the high data rate with low power, the great accuracy in location tracking and no additional requirements for repeater nodes compared with the other two schemes, make UWB technology very promising for underground mine communication and monitoring [29,30].

Above all, constructing wireless multimedia monitoring network for underground coalmine based on MB-OFDM-UWB wireless transmission technology is a promising and ideal way to realize coalmine multimedia surveillance, making full use of UWB’s characteristics of high speed, low power consumption and so on, and underground coalmine’s characteristic of open frequency resource.

As a new-generation wireless communication technique, the ultra-wide band (UWB) is the key technique to realize high speed wireless communication [31]. UWB provides wireless connection solutions for consumptive electronic devices. It enables a variety of applications to run on a general platform, realizing a high-speed and interoperable wireless multimedia communication. The data transmission rate of UWB is extremely high, from 100 Mbps to 500 Mbps within ten meters, and can even reach the level of Gbps [32]. At the same time, UWB also has varieties of characteristics, such as high spectrum efficiency, robustness to multi-path, low transmit power, high system security, simple structure, low costs and so on.

The initial UWB technique did not use the carrier but the ns-ps level non-sinusoidal pulses with very short duration for data transmission [31]. Because of the high peak energy of narrow pulse, UWB using narrow pulses is not suitable for dangerous environments, such as the underground coalmine [33]. At present, two mainstream solutions of UWB are Multi-Band Orthogonal Frequency-Division Multiplexing (MB-OFDM) and Direct-Sequence Code Division Multiple Access (DS-CDMA). These two solutions are both at the bottom layer i.e., physical layer of the whole UWB structure, but each has its own characteristics [34]. The MB-OFDM solution, led by Intel, Texas Instruments (TI) and other enterprises, and supported by the WiMedia Alliance which is made up by more than 200 enterprises all over the world including HP, Microsoft, Nokia, and Philips, is especially suitable for a variety of wireless multimedia application. On the other hand, the DS-CDMA solution, also known as the DS-UWB solution, is mainly led by Motorola and Freescale, and pushed by the UWB forum. Based on the MB-OFDM solution, the WiMedia UWB platform has become the first international UWB commercial standard.

The MB-OFDM-UWB solution divides the whole bandwidth into several sub-bands, which will enable data processing over a much narrower bandwidth. Additionally, it has also inherited the advantages of the multi-carrier modulation, such as the abilities to efficiently eliminate the inter-symbol interference (ISI) owing to multi-path propagation, and the complexity of the receivers. Therefore, the MB-OFDM-UWB solution is well-suited for the multi-path propagation environment like coalmine. The communication environment for underground coalmine is a typical representation of the confined space. The walls of laneways bring in plenty of refraction and scattering paths, resulting in the complex multi-path propagation of signals in laneways [35]. The delay spread caused by multi-path propagation can be seen as a selective fading of frequency in the frequency domain. In order to overcome the influence of different deep fading which is corresponding to different frequency, the whole channel is divided into several sub-channels in the frequency domain, turning the wide band frequency selective fading channel into different narrow band flat fading sub-channels. In Table 2, a comparison between MB-OFDM-UWB and the other two UWB techniques is summarized [36]. From the comparison, we can know that MB-OFDM-UWB outperforms in interference and also has great performance in other aspects. In the article [37,38], through the in-depth analysis of underground coalmine channel environment and the characteristic of multi-carrier modulation, our group confirms that OFDM is a suitable ultra-wideband technique for communication in underground coalmine environment. 

Consequently, choosing the modulation scheme of MB-OFDM-UWB as the wireless transmission mode for wireless multimedia sensors in the underground coalmine environment (that is, constructing WMSNs for underground coalmine based on MB-OFDM-UWB), can realize the integration of video surveillance for coalmine safety, wireless voice communication, underground environment monitoring and personnel positioning. Moreover, it will become a great means to promote the abilities of the coalmine safety monitoring, the early warning, and the rescue and relief of disasters.

## 3. Key Supporting Technologies for the WMSNs

**Figure 4:**
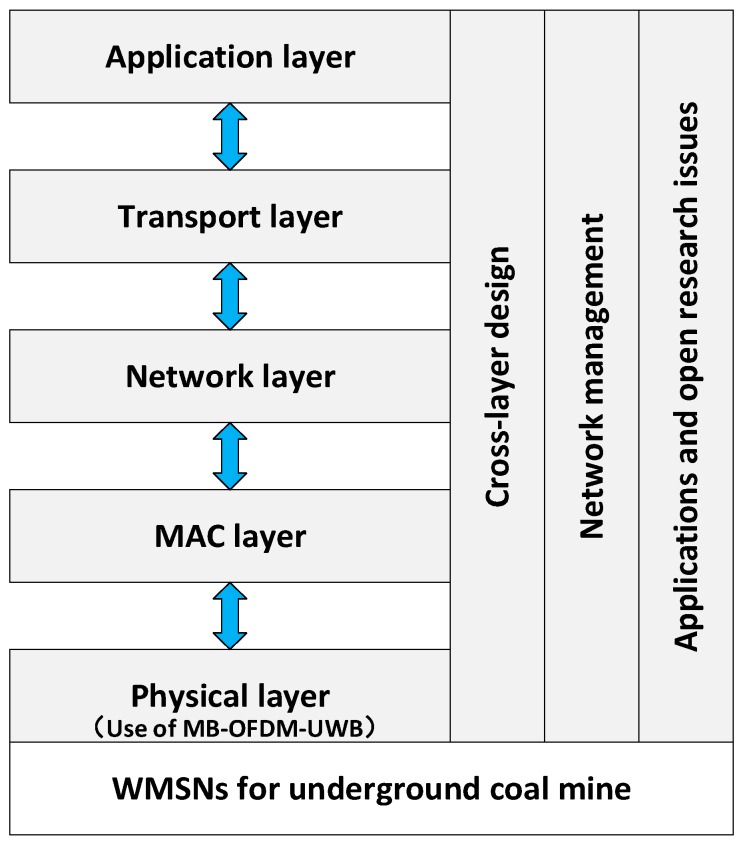
Main research issues for underground coalmine WMSNs.

The overall design of the WMSNs for underground coalmine is introduced in the previous section and the main focus is on the modulation scheme in physical layer. As for the WMSNs, there are some technologies which are also very important for its performance in underground coalmine. These technologies are called key supporting techniques, mainly including coverage-enhancing algorithms, media access control (MAC), routing mechanism, cross-layer optimization, and video encoding. All these key supporting techniques have been discussed to construct an integrated monitoring system based on the WMSNs for underground coalmine. Before further discussion of each section, a block diagram organized in layer-specific structure and giving the main research issues for coalmines is shown in Figure 4.

### 3.1. Coverage-Enhancing Algorithms

Due to the extreme and changing conditions of coalmines, deploying conventional wired networks is difficult and costly. For WMSNs, a successful deployment could enable tremendous savings in operational expenses because of enhanced communications and quality-of-service (QoS). Nevertheless, a hostile radio frequency environment (e.g., non-line-of-sight and multipath problems caused by heavy moving machinery and rock faces) and interference generated by heavy equipment are still challenges for engineers when considering deployment or coverage problems.

Coverage-enhancing issues, which are about how to deploy sensor nodes to maximize the coverage range with the guarantee of QoS, are basic problems for researching WMSNs [39]. Measuring the coverage performance of the network can help us find monitoring holes, thus guiding us to adjust the deployment of sensor nodes or add new nodes to improve the coverage performance of the system. Therefore, it is not simply an issue of deployment, but an issue concerning about QoS.

Based on the monitoring requirements, the sensor nodes of the WMSNs for underground coalmine can be classified into three types: video or image sensors, voice communication sensors and environment parameter monitoring sensors. Thus, the WMSNs for underground coalmine, made up by these sensors, are heterogeneous and multifunctional coverage networks. Currently, the research work on the coverage issues of WMSNs is concentrating on coverage-enhancing algorithms of homogeneous networks. Area coverage, target coverage, barrier coverage and connectivity coverage are main topics for the coverage issues. The coverage issue of heterogeneous networks, by contrast, is more difficult for research because we should consider the heterogeneous characteristics of sensor networks such as the different sensing models, the different information content of monitoring data and the difference between sensors’ resources or capacities [40]. Further studies are still necessary for the coverage issue of heterogeneous networks, considering that the existing research achievements of that are still less. 

The deployment problem of the monitoring sub-net mainly lies in the video surveillance sensors and environment parameter monitoring sensors. The voice communication sensors can be deployed according to the requirements of voice communication, thus not being our focus. Owing to the difference between the functions and the qualities of these two sensors, research work should be conducted separately. 

**Figure 5:**
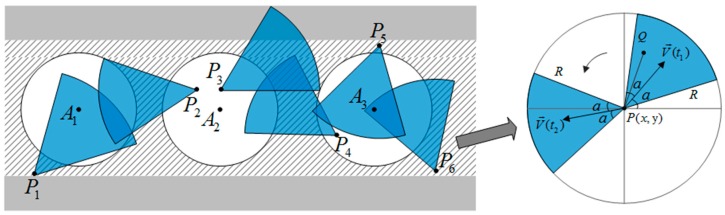
Effective coverage issue for WMSNs of coalmine and the directional adjustable sensing model.

Preliminary research work on the deployment problem of WMSNs in confined space like underground coalmine have been shown in [7,8]. As shown in Figure 5, Han and Yang [7] defined the effective coverage issue for underground coalmine WMSNs, considering characteristics of underground mine roadways and sensing models, and then enhanced the coverage performance by using improved virtual potential field method. Although the research work was aimed at homogeneous WMSNs, it still makes sense since the QoS of underground coalmine is boosted by focusing on coverage issues. In short, the problem of combining video surveillance sensors and environment parameter monitoring sensors to form an optimized data forwarding network structure under the requirements of coverage and QoS, need further studies for the sake of improving the efficiency and performance of networks. Providing safety guarantee and full-scale monitoring and communication service are the basic requirements for coverage issues.

### 3.2. MAC Protocols

Guaranteeing the bandwidth for transmitting multimedia data over wireless links, while ensuring the high transmission quality with limited sensor capabilities, are key factors for the network design of WMSNs. Media access control protocols, as the direct controller of sending and receiving all the data packets and the controlling packets over wireless channels, define the regulations of how to share media between the sensors to ensure the satisfactory network performance. Therefore, applying MAC protocols to efficiently use wireless channels are one of the crucial factors for guaranteeing the communication of WMSNs.

MAC protocols for WMSNs can be mainly categorized into three types: contention-based protocols, contention-free single channel protocols, and contention-free multi-channel protocols [4,41]. Contention-based protocols, using random timers and a carrier sense mechanism to avoid collisions, generally cannot guarantee the real-time requirements and support multimedia real-time data transmission. On the other hand, the main idea of contention-free protocols is assigning non-interference time slots or channels for each sensor, thus avoiding the collisions caused by sharing the same wireless channel. Contention-free protocols can achieve high link-level throughput and fairness. Unfortunately, most of the existing protocols, are not designed or improved for multimedia services transmission to offer the guarantee of QoS.

Some of the existing studies [11,42,43,44] on solving the MAC issues in coalmine WSNs do not take the real-time requirement into consideration, since only scalar data flow in the networks and the functionality of emergency multimedia communication may not be included in the initial design. Contention-based protocols were adopted in these works. To the author’s knowledge, there is little information available in the literature regarding MAC protocols for coalmine WMSNs; thus, further studies are still essential.

Recent years have seen many newly designed MAC protocols for WMSN that made effort to support multimedia services and satisfy the QoS requirements of users. In the paper [45], a new QoS-based sensory MAC protocol, which not only adapts to application-oriented QoS, but also attempts to conserve energy without violating QoS-constraints, is proposed. In comparison to other existing sensory MAC protocols, the proposed protocol is capable of providing lower delay and better throughput, at the cost of reasonable energy consumption. 

When designing MAC protocols for coalmine WMSNs, tradeoffs between the additional overhead of contention-free protocols and the energy savings realized through collision elimination of contention-based protocols need to be explored to definitively ascertain whether a single MAC protocol or a hybrid one should be used [46]. Meanwhile, the real-time requirement is also an important factor in WMSNs since there are more multimedia applications in the network. Therefore, according to the structure characteristics of the underground coalmine WMSNs, and the ways of channel assignment for MB-OFDM-UWB and factors mentioned above, further research work need to be done to design efficient MAC protocols that adapt to the QoS requirements of WMSNs for underground coalmine.

### 3.3. Routing Protocols

Just like traditional WSNs, the routing protocols, whose function is establishing and maintaining data transmission paths between any two nodes that need to communicate with each other, are also key part of researching WMSNs. However, the design of the routing mechanism for WMSNs is quite different from that for traditional WSNs in the following aspects:

(1) QoS: Most of the routing protocols for traditional WSNs mainly aim at minimizing the energy consumption, but introducing the multimedia contents like videos and images into WMSNs makes QoS a crucial issue. When designing routing protocols, not only the energy consumption, scalability and robustness of the network, but also real-time and reliable transmission of multimedia data need to be taken into consideration.
(2) Sensing models: Sensors in WMSNs are universally directional, e.g., video sensors, which make the sensing models or coverage models distinct from those of traditional WSNs. Distinctions in sensing models or coverage models will directly affect the design of network structure and routing protocols.
(3) Data aggregation: For the sake of reducing the amount of data and lowing power consumption, a majority of the routing protocols for WSNs have adopted data aggregation mechanism. In WMSNs, sensors nodes have strong directionality and transmits multimedia data which are information-rich and time-sensitive. The redundancy and the relevance of multimedia data need to be considered when aggregating data.

At present, based on the design principles of QoS guarantee, energy minimization or network lifetime maximization, stateless routing and so on, routing protocols of WMSNs including SPEED [47], MPMPS (Multi­priority Multi­path Selection) [48], REAR (Real-time and Energy Aware Routing) [49], and ReInForM (Reliable Information Forwarding using Multi-Paths) [50] have been proposed. 

Among these, SPEED is a location-based, stateless and real-time routing protocol for sensor networks, whose major object is to provide end-to-end soft real-time guarantee for the data streams with real-time requirements. Nonetheless, the protocol has not taken the energy characteristic of wireless networks or nodes into account. The MPMPS protocol is a WMSNs based routing protocol with multi-priority multi-path selection for video streaming. The core idea of MPMPS is designing multi-paths to increase the bandwidth, and decomposing video streams into audio streams and image streams. Higher priorities are assigned to important data streams which will occupy the paths with higher bandwidth and smaller time delay, and different paths are used depending on the levels of priority. In short, the features of MPMPS are decomposing video streams into sub-streams with different priorities and using multi-paths to efficiently increase the bandwidth. However, energy consumption is also not taken into consideration, and the process of decomposing or composing video streams brings in some time delay and high energy consumption. REAR is an energy-aware real-time routing protocol for WMSNs. The aim of this protocol is solving the real-time routing problem with the limitation of energy and QoS requirements. The superiorities of REAR are introducing metadata to establish multi-path routing for reducing the energy consumption, and constructing a cost function which trades off between energy and delay to evaluate the consumption of the transmission links, making REAR suitable for the QoS routing problem of WMSNs. Nevertheless, in the case of streaming applications, the metadata for streaming data can itself be very huge in mount and result in extra energy consumption [49]. In addition, the metadata may sometimes discard some useful information got from different viewing perspectives which cannot be simply overlooked. ReInForM, which provides desired reliability in data delivery based on the priorities of packets, uses redundant copies of a packet to increase its end-to-end probability of data delivery, but it only focuses on the reliability issue of QoS domain and does not take energy consumption and timeliness into account.

As we can see, although the typical routing protocols for WMSNs involve QoS issues, there are still defects in them, affecting their adaptability to multimedia data transmission in WMSNs. For the scenario of underground coalmines, link costs and network topology can vary over time. Therefore, the network should be aware of these changes and handle them quickly. Besides, WMSNs for coalmine will both act as a data collector and an emergency rescuer, and this may require the network to be suitable for time-sensitive applications while remaining power efficient. Thus, the designing of routing protocols for underground coalmine WMSNs should be based on the characteristics of network structure, the channel allocation scheme for MB-OFDM-UWB and the features of service for underground coalmine WMSNs. The protocols should be with QoS guarantee and good scalability, enabling them to adapt to the requirements of being real-time and reliable for the wireless video surveillance, wireless voice communication and underground environment monitoring.

### 3.4. Cross-Layer Design 

In traditional WSNs, the transmission strategies supporting multimedia streams, proposed by different layers, are generally confined to one single layer and ignore the interaction between layers. The network resource is restricted mutually by the physical layer, MAC layer and network layer: in physical layer, the interference at the receivers affect the multi access of nodes in wireless channels; the bandwidth for forwarding nodes assigned by MAC layer, has influence on the performance of detecting useful signals in physical layer; low bandwidth and high packet delays introduced by transmission schedules compel networks to change their routing, and the selection of routing protocols alter link scheduling schemes, thus affecting the performance of MAC layer. Therefore, cross-layer design is needed to improve the network performance, such as QoS, energy consumption and channel utilization rate.

The cross-layer design of WMSNs is a set of methodologies which combine the optimizing strategies of different layers and make use of potential improvements of exchanging information between different layers of the communication stack, to efficiently transmit multimedia data streams, though with the constraints of network resource and QoS. In [51], a new cross-layer communication architecture based on the time-hopping impulse radio ultra-wide band (TH-IR-UWB) technology is introduced, whose objective is to reliably and flexibly deliver QoS to heterogeneous applications in WMSNs, by leveraging and controlling interactions among different layers of the protocol stack according to applications requirements. In [52], a novel cross-layer framework for QoS support in WMSNs is proposed, which maximizes the capacity of the deployed network to enhance the number of video sources by means of mapping application requirements on joint operations of application, network, link and MAC layers to achieve the desired QoS.

Hence, according to the characteristics of multimedia content and applications for underground coalmine WMSNs, adopting cross-layer designing techniques will significantly enhance the performance of underground coalmine WMSNs. For instance, the coal dust concentration collected for application layer, can also be used by lower layers, to adjust radio output power to eliminate the interference incurred by coal dust and choose better routes. On the contrary, the channel properties can sometimes reflect the environmental parameters. For prolonging network lifetime, routing protocols will generally select the links with lower transmit powers. This information is collected by the physical layer, processed by cross-layer controller and utilized by the network layer, thus achieving power savings. 

With the object to make cross-layer designing techniques more practical, the models for cross-layer design can follow the principle of using a cross-layer controller to uniformly manage the operations and interactions among different layers of the protocol stack, although the network structure is still a hierarchical one. To minimize energy consumption and offer QoS guarantee for multimedia streams, the optimizing strategy for coding rate, data rate, and access control and so on, should alter in the light of the status of independent functional modules like UWB transceiver, source or channel encoder, and QoS scheduler.

### 3.5. Multimedia Encoding in Application Layer

Multimedia encoding techniques are important techniques in WMSNs which are directly related to video surveillance. The basic objectives of their design are as follows: (1) achieve high compression efficiency to effectively limit bandwidth and energy consumption, with the guarantee for the quality of video surveillance; (2) have low complexity and power consumption to enable multimedia encoders to be embedded in sensor devices and to prolong the lifetime of sensor nodes; and (3) provide robust and error-resilient coding of source data. For typical state-of-the-art video compression standards, such as MPEG, H.263 and H.264, which employ the motion estimation functionality at encoders, the encoder is five to ten times more complex than the decoder [53]. Thus, the low-complexity and low-power requirements for the sensor nodes in WMSNs could not be satisfied.

Distributed source coding (DSC) adopts the technique of combining intra-frame encoding with inter-frame decoding, which leverages knowledge of the source statistics at the decoder and reduces the complexity of coding at the encoder, making itself extremely suitable for WMSNs [54]. DSC has the characteristics of easy encoding and complex decoding, and are highly complementary with the traditional video encoding whose characteristics are reversed. In recent years, the research focus is on the distributed source encoding techniques based on Wyner–Ziv encoding, such as Pixel-domain Wyner–Ziv encoding and Transform-domain Wyner–Ziv encoding [53,55]. A rate compatible punctured turbo code (RCPT), which is often used in Wyner–Ziv encoder and decoder, is proposed in [56]. In [57], a distributed video coding method for constraining the number of feedback requests to a fixed maximum number of N requests for an entire Wyner-Ziv frame is proposed, achieving great trade-offs between time delay of the system and the complexity of encoding. Other research works on distributed source coding in sensor networks can be found in [54].

Though great effect has been achieved by these distributed video coding techniques, most of the recent research papers are still confined to discussing the theory of coding and decoding itself, leaving lots of problems to be explored and discussed in the area of combining the practical application of WMSNs. 

Since video distortions, felt by end users, depend on source coding and channel coding, multimedia encoding techniques for coalmine WMSNs should take the factors like channel coding techniques for coalmine environment into consideration. Besides, the environments of coalmines are generally with poor light and more coal dust, which have great influence on the quality of video surveillance, video streams in coalmines present different statistic features from traditional surficial applications, and these features should be utilized by encoding techniques. In addition, network topology may also effect the implementation of the Wyner-Ziv video coding algorithm [58]. 

Therefore, the video surveillance techniques should be associated with the MB-UWB wireless transmission technique, the transmission characteristics of wireless channels in coalmine tunnel, and the temporal and spatial relationship between video sensors in coalmine. Moreover, the distributed video coding techniques should be designed to meet the requirements of video surveillance for underground coalmine.

## 4. Other Research Issues

Although most of the key supporting technologies have been shown in the aforementioned layer-specific discussion, additional issues should be addressed to make the structure of underground coalmine WMSNs an integrated one. Some other important issues like UWB localization, video localization, and information processing and network management are considered in this section.

### 4.1. UWB Localization

Besides the application sceneries mentioned above, WMSNs for underground coalmine will also plays an important role in production automation, people or asset tracking, security assisting or rescuing and underground coalmine robot navigation. Localization techniques for underground coalmine include the techniques for locating wireless sensor node itself and locating monitored targets, where the latter one is especially essential for underground coalmine applications such as target monitoring, robot navigation, and motion analysis for miners.

For the self-localization problem of sensor nodes, since UWB technique is adopted in the physical layer of the communication stack, thus the problem turns to a UWB based localization problem of sensor nodes. As for the UWB localization technique which adopts impulse radio (IR), centimeter-level or even millimeter-level ranging accuracy is theoretically possible, due to the extremely short duration of transmitted pulses and the strong ability of time-resolving. Specifically, the UWB ranging system based on impulse radio technology regularly figures out the distance between sending and receiving end, by estimating the time of arrival (TOA) of the earliest component of received signals. In recent years, IR-UWB based TOA position estimation algorithms have been well studied [59,60], including the coherent TOA estimation position algorithm in which high sampling rate and high-precision match filter (MF) are used, and the no-coherent energy-collection TOA position estimation algorithm which adopts low sampling rate and lower complexity.

Nevertheless, the algorithms are mainly concentrating on IR-UWB technique, while sensor nodes in underground coalmine WMSNs use MB-UWB technique in their physical layer which is quite different from IR-UWB. Thus, the current IR-UWB localization techniques could not be applied in MB-UWB based system. The localization problem for the sensor nodes in underground coalmine should considering the structure of network and MB-UWB techniques in physical layer, leading to corresponding localization algorithms.

### 4.2. Target Localization

The sensing model for video sensors in WMSNs is based on directional sensing model which utilizes perspective projection relationship of cameras, which differs remarkably from the omni-directional sensing model for traditional WSNs. Thus, there are significant differences between the target localization algorithms of the two. In recent years, the target localization problem has received considerable attention and research work in the area of WMSNs. 

**Figure 6:**
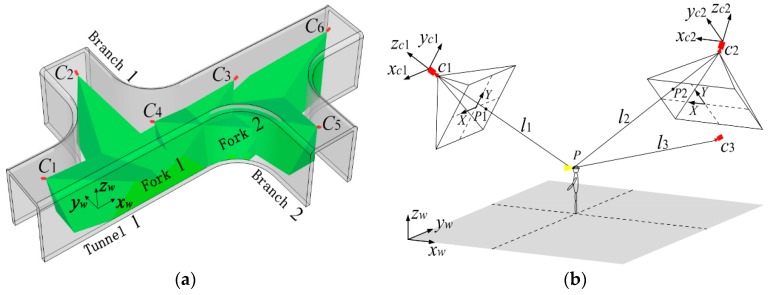
Video collaborative localization of miner’s lamp based on WMSNs: (**a**) localization scene; and (**b**) camera vision imaging model.

An object localization scheme for WMSNs, which can be run on a single camera node by utilizing the size of the object on the frame obtained from frame differencing and the monocular vision imaging model, has been proposed in [61]. In [62], the authors have presented a cooperative target localization algorithm which can achieve a good trade-off between energy consumption and accuracy of localization, on the issue of node selection for target localization in WMSNs. Li et al. [63] has raised a single-node and a double-node target localization algorithm used in simplified calibration scenes, according to the characteristics of multi-mode cooperation in WMSNs. You et al. [64] presented a video collaborative localization algorithm which uses miners’ lamps as the key feature and the camera vision imaging model, to estimate the real location of miner for coalmine scene. As shown in Figure 6, the localization process is performed collaboratively by three video sensors. The research and experimental work of [64] indicates a new way for localization and target tracking in coalmine. Consequently, the physical appearance and outfit of miners provide extra information for analyzing their locations and behaviors, which further help to meet the higher safety standards and satisfy the growing demand for multimedia data. Just as human beings use eyes to track and confirm targets, target localization with WMSNs endows underground mines with “eyes”. Combining with the UWB localization mentioned in the previous section, monitoring system for underground coalmines can achieve both good location accuracy and intuitive visual information.

From the existing research work, it can be seen that the study on target localization techniques for WMSNs should be related with specific conditions in the acquiring methods, contents, types and quantity of information. Likewise, the target localization techniques for underground coalmine WMSNs should also take the network structure, characteristics of monitoring scenes, MB-UWB wireless transmission mode, and processing capacity of sensor nodes into consideration, as well as be designed by combining a distributed source coding scheme and a target capturing scheme. 

### 4.3. Information Processing

With the limitations in energy, processing capacity and other resources of sensor nodes, introducing information processing techniques into the acquiring, transmitting and displaying information processing techniques in WMSNs is an effective way to lift the QoS performance of multimedia service. Among these information processing techniques, compression coding, filtering and data fusion are the most common ones.

Compression coding techniques compress image, audio and video streams which are tremendous in data amount, saving the bandwidth resource of network while providing high-quality multimedia service. Filtering techniques can filter out parts of data which are not interesting for observers and not valuable for monitoring results from raw sensing data, thus gaining useful monitoring information which are small in data quantity. In energy-limited WMSNs, the research for these information processing techniques would be particularly important. Apart from compression coding and filtering, data fusion also plays an important role, which is mainly shown in saving the resource of network and enhancing the accuracy of collected data. 

In [65], for video sensor networks, an image fusion algorithm based on visual correlation is proposed. Given the limited resource constraints on each single video sensor and the redundant visual information among multiple video sensors, the algorithm partitions a sensing task into the two highly correlated video sensors and utilizes the epipolar constraint to fuse the received multiple partial images and to reconstruct a complete visual scene. Shown by experiments, the algorithm can not only reduce the transmission workload and save network energy, but also conduct the visual monitoring task in an effective way. Nevertheless, the method works on the system level, without considering the pixel-level redundancy in lower layer and video compression techniques. In addition, the assumed conditions in reconstructing a scene restrict the algorithm from being applied in the scene which has different hierarchy or depth. On the basis of YUV color space and the view correlation of adjacent nodes, Wang et al. [66] has come up with an image fusion method that assigns monitor task to three video sensor nodes that are highly correlated. Then, with the depth information model, adaptive quadtree partitioning and space transform, the luminance and chrominance parts of decoded data are fused, which realizes the reconstruction of the color image of the scene. The method has achieved good tradeoffs between the storage of video sensors, transmission cost and scene monitoring quality. For the sake of generating high-resolution images with wide field of view under constraint network resource, Xiong et al. [67] utilizes the redundancy of visual information among multiple video sensors, and stitches two or more images which have overlapped regions together to make a wide-angle high-resolution one. Thus, the requirements of advanced applications in video surveillance can be satisfied, and the network load can be reduced which thereby prolongs network lifetime. In brief, these algorithms involve multiple information processing techniques and achieve evident effects.

The WMSNs for underground coalmine normally consist of a large number of video, audio and scalar sensor nodes. Given a source of monitoring data, different applications in a network may require disparate information (e.g., for video surveillance, video streams are needed while for coalmine environment monitoring, scalar data is collected). In addition, some information, such as signals for detecting high concentration of flammable gas, is with top priorities and needs to be reported directly to the ground monitoring center, while other information like air humidity may be queried and processed with normal periods and priorities. Accordingly, it is necessary to deal with these application-specific querying and processing, and to develop distributed filtering and in-network processing architectures, to allow real-time retrieval of useful information [4]. Besides, architectures that allow data fusion or other complex in-network processing operations should be proposed. 

For the purpose of gaining useful information which is more accurate and concise, information processing techniques in different level, aiming at different network structure, MB-UWB wireless transmission mode and specific application requirements, should be proposed. By introducing information processing, the overall monitoring performance of coalmine WMSNs will be sufficiently improved.

### 4.4. Network Management

Sensor nodes in underground coalmine WMSNs are large in numbers and restricted in resource, and the environment condition of monitoring regions is generally harsh. If no network management strategy is conducted in the process of network planning, deployment and maintenance, it will be difficult to realize effective monitoring on monitoring regions. The reason is that once the sensor network is deployed, maintaining a network utterly by man seems really hard and even impossible. Electromagnetic interference, different kinds of adverse environment conditions and limited energy of sensors all can be factors that lead to breakdowns or damages in sensor devices, thus making the whole network prone to malfunctions or even crashes. 

Network management for sensor network aims to realize the following requirements: (1) develop effective management strategies in light of the characteristics of sensor network; (2) monitor various status parameters in real time; and (3) realize self-judgment, self-maintenance and self-determination. Accordingly, by network management, sufficient utilization of network resource can be realized, and we can also provide reliable QoS for applications and satisfy the service requirements of multimedia monitoring in underground coalmine WMSNs. 

Typical framework for network management in WSNs includes BOSS [68] and MANNA [69]. BOSS (short for Bridge of the SensorS) can be regarded as an intermediary which is able to interpret and transfer messages between the UPnP controllers and the non-UPnP sensor nodes to be managed, while MANNA offer a unified framework for the strategy-based network management of WSNs [70,71]. Network management protocols for WSNs mainly includes TopDisc and STREAM in sNMP which are used for extracting topology information of the network, the zone flooding scheme used in RRP, the Drip protocol introduced by SNMS, and FlexiMAC in WinMS [72,73]. 

In [11], the authors designed two working modes, namely the periodic inspection mode and interrupt service mode, for a coalmine environment monitoring system based on WSNs. The two modes correspond to the reactive monitoring and proactive monitoring in the management reactivity. Then, based on the data or events collected by the network, different management tasks can be distributed in the network. In addition, topology management, location management, energy management and fault management are also adopted in their system, ensuring the normal operation of coalmine WSNs. However, for coalmine WMSNs, the unreliable communications incurred by harsh environment of coalmine and various amounts and types of data brought by WMSNs, make it more challenging to design comprehensive frameworks and protocols for network management in coalmine WMSNs.

However, the existing frameworks and protocols for network management in WSNs have not been fully functional, and the research work on network management in WMSNs is still few in number. Thus, in order to guarantee the efficient configuration, operation, administration, and maintenance of underground coalmine WMSNs, efficient frameworks for network management and corresponding protocols should be proposed, combined with specific applications of networks.

## 5. Discussion for Future Research Directions

While we expect smart coalmines to continue to evolve in the future, it is necessary to consider the most robust and reliable technology to facilitate the underground communication in wireless multimedia sensor network. In the sections above, we highlight the superiority of MB-OFDM-UWB in data rate, normalized energy consumption, robustness against fading, immunity to multipath and localization performance, and stress choosing it in underground WMSNs as it performs better than ZigBee and Wi-Fi. 

It should be stressed, however, that underground mines have many different types of communication such as ground, in-mine, through-the-earth, and disaster communications, and it is difficult to present a single-structure system that can provide solutions to all the applications simultaneously. Except for the research of applying MB-OFDM-UWB into underground coalmine WMSNs, we are still paying our attention to the hybrid schemes which will use multiple short-range wireless transmission techniques. 

The emerging standard IEEE 802.11ac [74] has brought some new features like providing high-throughput to the 802.11 family, but the industrial applications of it, especially for coalmine WMSN, are almost blank. In addition, the techniques of multiple-input multiple-output (MIMO) facilitate the 802.11ac with higher speed. Thus, combining 802.11ac with UWB maybe a good choice to establish a hybrid WMSN that supports multimedia information transmission since both of them may deliver gigabit wireless. In addition, some antenna selection techniques such as using multiple antennas can also be utilized in UWB to solve the problem of data rates, transmission range, and interference [75].

As Cross-layer design is emphasized in Section 3.4, the latest findings from different layers, e.g., wave propagation in the specific type of mine tunnel, are related to the system performance or complexity. The UWB technology is different to the classical narrowband communication in many aspects and thus there are still underutilized potentialities in underground mine applications despite recent drawbacks caused by the market meltdown and delays in delivering new products. In addition, some new concepts like mashup middleware [76], internet of things (IOT) and cloud computing [77] are introduced into the sensor network for underground coalmines which help to increase the operational efficiency and improve the overall level of mining safety. 

## 6. Conclusions

Since the systems for coalmine safety monitoring, early warning and disaster relief have urgent demands for wireless video surveillance, wireless voice communication, underground environment monitoring and personnel positioning, this paper presented a framework for constructing a wireless multimedia sensor network for underground coalmines that integrates all these functions. The framework and related discussion filled the gap of absence of efficient and comprehensive structure for designing WMSNs for coalmine.

Overall design of WMSNs was presented first, and we discussed in detail the main design challenges, in which MB-OFDM-UWB solution was selected in physical layer. We initially compared the schemes of ZigBee and Wi-Fi with the UWB scheme, and found that UWB can solve both constraints of bandwidth and energy consumption. Then, we further analyzed the several UWB solutions and stress the superiority of MB-OFDM-UWB scheme. The selection of MB-OFDM-UWB wireless transmission solution was based on the characteristics of underground coalmine, and that is different from other schemes for WMSNs which are based on DS-UWB solution. MB-OFDM-UWB transmission solution, together with the characteristics of applications and environments in underground coalmines, was the cornerstone of future research and discussion work. Layer-specific structure was used to discuss the key supporting technologies for underground coalmine WMSNs. Some promising open research areas were also presented, indicating a possible direction for future works.

## Figures and Tables

**Table 1:** Comparison of UWB, Wi-Fi and ZigBee.

Parameters	UWB	Wi-Fi	ZigBee
IEEE Standardization	802.15.3a ^1^	802.11	802.15.4
Maximum Transmission distance	Up to 10 m	200 m	10–100 m
Frequency range	3.1–10.6 GHz	2.4 GHz, 5 GHz	868/915 MHz, 2.4 GHz
Data rate	480 Mbps	250 Mbps	250 kb/s
Energy consumption	High	High	Low
Normalized energy consumption	Low	Low	High
Complexity	Medium-High	High	Low

^1^ Unapproved draft.

**Table 2:** Comparison summary of UWB techniques.

Method	Name	Interference	Robustness to Multipath	Performance	Complexity	Achievable Range-Date Rate
Multiband	MB-OFDM	+++	++	+++	+	+++
IR	DS-UWB	++	++	+++	++	+++
IR	TH-UWB	++	++	++	++	++

Legend levels: Excellent: +++; Very Good: ++; Good: +.
